# Observational multicenter study to evaluate the prevalence and prognosis of subclinical atheromatosis in a Spanish chronic kidney disease cohort: baseline data from the NEFRONA study

**DOI:** 10.1186/1471-2369-15-168

**Published:** 2014-10-18

**Authors:** David Arroyo, Angels Betriu, Montserrat Martinez-Alonso, Teresa Vidal, Jose Manuel Valdivielso, Elvira Fernández, on behalf of the investigators from the NEFRONA study

**Affiliations:** Nephrology Department, Hospital Universitari Arnau de Vilanova, Avda. Rovira Roure 80, 25198 Lleida, Spain; Unit for Detection and Treatment of Atherotrombotic Disease (UDETMA), Nephrology Department, Hospital Universitari Arnau de Vilanova, Avda. Rovira Roure 80, 25198 Lleida, Spain; Statistics Department, IRB-Lleida, Hospital Universitari Arnau de Vilanova, Avda. Rovira Roure 80, 25198 Lleida, Spain; Experimental Nephrology Laboratory, IRB-Lleida, Hospital Universitari Arnau de Vilanova, Avda. Rovira Roure 80, 25198 Lleida, Spain; Spanish Renal Investigation Network – RedInRen, Instituto de Salud Carlos III, Madrid, Spain

**Keywords:** Chronic kidney disease, Cardiovascular disease, Atheromatosis, Vascular calcification, Intima-media thickness, Ankle-brachial index

## Abstract

**Background:**

Cardiovascular events (CVE) are more prevalent in chronic kidney disease (CKD) than in general population, being the main cause of morbimortality. Specific risk factors related to CKD have been suggested, because traditional factors do not fully explain this increase in cardiovascular disease rates. However, the role of atheromatosis, its pathogenesis and evolution are still unclear. The potential use of diagnostic tests to detect subclinical atheromatosis has to be determined.

**Methods:**

NEFRONA is a prospective multicenter cohort study. 2445 CKD subjects were enrolled from 81 Spanish hospitals and dialysis clinics, from 2010 to 2012. Eligibility criteria included: 18 to 74 years old, CKD stage 3 or higher, and no previous CVE. 559 non-CKD controls were also recruited. Demographical, clinical and analytical data were collected. Carotid and femoral ultrasounds were performed by a single trained team to measure carotid intima-media thickness (cIMT) and detect atheromatous plaques. Ankle-brachial index (ABI) was measured.

**Results:**

Differences in age, sex and prevalence and control of cardiovascular risk factors were found between controls and CKD patients. These differences are similar to those described in epidemiological studies.

No difference was found regarding cIMT between controls and CKD (when subjects with plaques in common carotid arteries were omitted); earlier CKD stages had higher values. CKD patients had a higher rate of atheromatous plaques, with no difference between stages in the unadjusted analysis. A group of patients had plaques in femoral arteries but were plaque-free in carotid arteries, and would have gone underdiagnosed without the femoral study. The percentage of pathologic ABI was higher in CKD, with higher prevalence in more advanced stages, and a higher rate of ABI >1.4 than <0.9, suggesting more vascular calcification.

**Conclusions:**

NEFRONA is the first large study describing the actual prevalence of subclinical atheromatosis across different CKD stages. There is a very high rate of atheromatous plaques and pathologic ABI in CKD. Prospective data will add important information to the pathogenesis and evolution of atheromatosis in CKD, compared to non-CKD subjects.

## Background

Chronic kidney disease (CKD) is a growing health problem, with increasing morbidity, mortality and monetary costs [1]. Despite the improvement in the treatment of its complications, patients with CKD in any stage continue to have a higher rate of cardiovascular events than subjects with normal renal function. In fact, cardiovascular disease (CVD) continues to be the first mortality cause in this population, and in many recent guidelines it has been stated as a cardiovascular risk factor itself, as severe as diabetes [2].

Important advances have been made in the knowledge of both general and specific cardiovascular risk factors in CKD. However, neither traditional risk factors or risk score charts for general population nor emerging risk biomarkers appear to be good enough predictors in this population [3–6]. It has been suggested that there is a different pathogenesis for CVD in advanced CKD, since most cardiovascular deaths appear to be from sudden deaths. These events have been related to possible arrhythmias or heart failure, related to heart remodelling and microischemia of the myocardium [7–9]. Besides, the effects of reverse epidemiology of many traditional risk factors and the appearance of different mechanisms are involved [10–12].

The presence of accelerated atheromatosis in CKD could also play a role in the higher cardiovascular mortality, in a process that has been termed accelerated vascular aging. However, there is still much to clarify regarding the process of atheromatosis in this group of patients, and the precise contribution of various risk factors. Many serum biomarkers are altered in CKD patients, and their prognostic value is currently under study [13, 14], but new tools for adequately assessing asymptomatic atheromatosis are required [15]. The application of diagnostic tests in CKD (i.e. vascular ultrasound or ankle-brachial index) to detect subclinical atheromatosis and adequately stratify cardiovascular risk has not yet been specifically addressed, although there are some initial promising data [16, 17].

The NEFRONA project is an observational multicenter prospective study designed to evaluate the prevalence and evolution of subclinical atheromatosis in CKD patients, as well as the contribution of vascular imaging for a more precise cardiovascular risk assessment [18, 19]. Baseline data of this study are presented in this article.

## Methods

### Study design and participants

NEFRONA is a prospective multicenter cohort study, in which 2445 CKD subjects were enrolled in 81 Spanish hospitals and dialysis clinics, from October 2010 to June 2012. Patients between 18 and 74 years of age were eligible if they had CKD stage 3 or higher as defined by current guidelines (glomerular filtration rate lower than 60 mL/min/1.73 m^2^ estimated using the 4-variable Modification of Diet in Renal Disease (MDRD) equation) [20]. Besides, 559 controls with an MDRD over 60 mL/min/1.73 m^2^ were recruited from Primary Care centers. Exclusion criteria for both groups included: active infections, pregnancy, life expectancy lower than 12 months, and history of cardiovascular events, carotid artery surgery or any organ transplantation.

The sample size was calculated based on the incidence of cardiovascular events described in the CKD population and depending on the stage of kidney disease [21]. The minimum sample required is based on the number of cases studied to obtain a minimum number of events (assumed to be homogeneously distributed throughout the follow-up period) to adjust the results of the multivariate analysis for 15 variables. We fixed type I and II errors to 5% (bilateral) and 10%, respectively. It was assumed a 25% loss of follow-up, and that both events and losses of follow-up will be proportionally distributed over the study period. Consequently, it was planned to include a total of 2661 patients (1325 stage 3 CKD, 713 stage 4 and 5 CKD, and 623 in dialysis). Furthermore, 843 subjects without renal disease were planned to be included to detect significant differences in the presence of plaques among patients for each stage of kidney disease and subjects without kidney disease (estimated from data provided by the Hospital Arnau de Vilanova, Lleida).

Patients were provided with and signed an informed consent. The local Ethics Committee of the Hospital Arnau de Vilanova approved the protocol.

### Clinical and laboratory data

Recruiting investigators completed a questionnaire with the patients’ clinical data, including family history of early cardiovascular disease, cardiovascular risk factors (such as smoking, diabetes, hypertension or dyslipidemia) and current medications. Anthropometrical data and vitals were obtained using standardized methods, as described in the study design [18]. Intact parathyroid hormone, 25-hydroxi-vitamin D and 1,25-hydroxi-vitamin D were all measured in a centralized laboratory.

Biochemical parameters were obtained from a routine fasting blood test taken no more than three months apart from the vascular explorations. In hemodialysis patients, samples were obtained before the second dialysis session of the week.

### Atherosclerosis assessment

Detailed and technical information on the methods to evaluate subclinical atherosclerosis has been previously published [18]. All studies were performed with a standardized protocol by three experienced itinerant teams including a nurse and a radiology technician. These teams also recorded anthropometrical parameters and collected blood samples to be stored in the REDinRen (Spanish Renal Research Network) centralized biobank for future biomarker studies.

Participants underwent a carotid and femoral ultrasound to measure carotid intima-media thickness (cIMT) and to evaluate the presence and characteristics of atheromatous plaques, defined as a cIMT lumen protrusion ≥1.5 mm, as recommended by the American Society of Echocardiography [22]. cIMT was recorded as the mean value between right and left common carotid intima-media thicknesses. There is no current consensus about the way to measure cIMT in patients with atheromatous plaques in common carotid arteries. We propose to use a right-truncated value of 1.5 mm in the presence of plaques in the corresponding artery and then computing cIMT as the mean value between right and left common-carotid arteries values (equivalent to the median of both values). Plaque presence in other carotid territories was not evaluated for cIMT measurement.

Plaque presence was evaluated in left and right sides, and in various territories (internal and common carotid arteries and carotid bulbs, and common and superficial femoral arteries).

Ankle-brachial index was measured using a protocolized method, and the modified ABI was recorded. This is the lowest value of the four available, and it was preferred because it is the measure with a higher sensitivity for cardiovascular risk assessment [23, 24]. A pathologic ABI is defined as a value <0.9, diagnostic of a limb ischemia, or >1.4, diagnostic of arterial incompressibility and stiffness, usually ascribed to vascular wall calcification.

An atherosclerosis score (AS) was originally designed and described in the study rationale article [18], according to these measures: AS0 (no atheromatosis), ABI >0.9 and cIMT <90^th^ percentile of reference values; AS1 (mild), ABI between 0.7-0.9 or cIMT ≥90^th^ percentile of reference values; AS2 (moderate), carotid plaque with stenosis <50-70%; and AS3 (severe), ABI <0.7 or carotid plaque with stenosis ≥70%.

### Statistical analysis

Statistical analysis was performed using SPSS software, version 17.0 (Chicago, Ill, USA) and R software [25]. Quantitative data are expressed as mean values ± standard deviation, while qualitative variables are given in absolute and relative frequencies. Normal distribution was assessed by the Kolmogorov-Smirnoff test. Χ^2^ and Fisher exact’s tests were used to compare categorical data, Student’s t-test and Mann-Whitney’s U-test for continuous data, ANOVA and Kruskal-Wallis’s H-test for comparison of several groups and Pearson and Spearman’s coefficients for numerical correlations. A significance level of 0.05 was accepted.

## Results

Main clinical and demographical data are summarized in Table 1. Table 2 includes information related to anthropometrical data and pharmacological treatments at enrolment, while Table 3 aggregates results of laboratory values. Data are presented for total groups and for different CKD stages. There are remarkable differences in age, sex and prevalence and control of cardiovascular risk factors between CKD patients and controls, but also between different CKD stages. These differences will presumably have an effect on subclinical atheromatosis.

**Table 1:** **Baseline demographic and clinical characteristics of the patients**

	Controls	CKD	CKD stage 3	CKD stages 4-5	CKD stage 5D	p value	p value
	n =559	n =2445	n =937	n =820	n =688	CKD vs controls	Between CKD groups
Male gender	298 (53.3)	1509 (61.7)	621 (66.3)	478 (58.3)	409 (59.4)	<0.001	<0.001
Age (years)	54.6 ± 11.5	57.9 ± 12.8	60.9 ± 11.4	58.7 ± 12.3	53.2 ± 13.8	<0.001	<0.001
**Clinical history**							
Familial history of CVD	62 (11.1)	213 (8.7)	93 (9.9)	73 (8.9)	47 (6.8)	NS	NS
Current smoker	109 (19.5)	474 (19.4)	177 (18.9)	158 (19.3)	175 (20.2)	NS	NS
Former smoker	228 (40.8)	862 (35.3)	343 (36.6)	279 (34.0)	204 (34.9)	NS	NS
Hypertension	197 (35.2)	2183 (89.3)	827 (88.3)	766 (93.4)	590 (85.8)	<0.001	<0.001
Diabetes mellitus	61 (10.9)	628 (25.7)	258 (27.5)	246 (30.0)	124 (18.0)	<0.001	<0.001
Dyslipidemia	196 (35.1)	1587 (64.9)	657 (70.1)	567 (69.1)	363 (52.8)	<0.001	<0.001
**CKD etiology**	N/A					N/A	<0.001
Vascular		471 (19.3)	272 (29.1)	128 (15.6)	71 (10.3)		
Glomerular		381 (15.6)	110 (11.8)	150 (18.3)	121 (17.6)		
Diabetic		351 (14.4)	126 (13.5)	145 (17.7)	80 (11.6)		
Tubulointerstitial		287 (11.7)	94 (10.4)	100 (12.2)	93 (13.5)		
Cystic		239 (9.8)	58 (6.2)	85 (10.4)	96 (14.0)		
Other		307 (12.5)	103 (10.6)	102 (12.4)	102 (14.8)		
Unknown		407 (16.7)	172 (18.4)	110 (13.4)	125 (18.2)		

Data expressed in number (%) or mean value ± standard deviation. *Abbreviations:* *CKD* chronic kidney disease, *CVD* cardiovascular disease, *NS* not significant, *N/A* not assessed.

**Table 2:** **Baseline anthropometrical data and treatments at enrolment**

	Controls	CKD	CKD stage 3	CKD stages 4-5	CKD stage 5D	p value	p value
	n =559	n =2445	n =937	n =820	n =688	CKD vs controls	Between CKD groups
**Anthropometrical data**							
SBP (mmHg)	133.2 ± 17.7	142.9 ± 22.0	142.9 ± 20.2	146.7 ± 22.1	138.4 ± 23.4	<0.001	<0.001
DBP (mmHg)	80.0 ± 9.7	81.3 ± 11.9	81.6 ± 10.2	82.2 ± 11.4	80.0 ± 14.2	0.010	0.005
Pulse pressure (mmHg)	53.2 ± 13.0	61.6 ± 18.3	61.3 ± 16.9	64.5 ± 19.4	58.4 ± 18.3	<0.001	<0.001
BMI (Kg/m^2^)	28.1 ± 4.5	28.3 ± 5.2	29.2 ± 4.9	28.8 ± 5.5	26.5 ± 4.9	NS	<0.001
Abdominal obesity (%)*	47.8	49.6	58.9	49.3	34.4^a^	NS	<0.001
**Pharmacological treatments**							
Antihypertensive (%)	34.5	88.1	91.8	95.6	74.3	<0.001	<0.001
Hypolipidemic (%)	27.4	64.4	67.6	70.7	52.5	<0.001	<0.001
Antidiabetic (%)	9.5	21.9	23.5	25.2	15.8	<0.001	<0.001
Antiaggregation (%)	5.9	24.8	23.6	23.8	27.6	<0.001	NS
Anticoagulation (%)	0.5	3.1	3.2	3.0	3.2	<0.001	NS
Vitamin D (%)	N/A	38.7	18.0	50.4	53.1	N/A	<0.001
Of which active (%)	N/A	32.1	12.0	42.8	46.9	N/A	<0.001
Of wich native (%)	N/A	10.2	7.0	13.6	10.6	N/A	<0.001
Phosphate binders (%)	N/A	35.7	5.7	31.1	82.0	N/A	<0.001
Non-calcium based (%)	N/A	22.2	1.0	14.7	60.1	N/A	<0.001
Calcium based (%)	N/A	21.1	4.9	20.4	43.8	N/A	<0.001
ESA (%)	N/A	35.6	5.7	32.2	80.4	N/A	<0.001
Intravenous iron (%)	N/A	18.7	1.7	8.2	54.5	N/A	<0.001

Data expressed as percentage or mean value ± standard deviation. *Abdominal obesity defined as waist circumference >88 cm in women and >102 cm in men. ^a^Waist circumference was not measured in peritoneal dialysis patients. *Abbreviations:* *CKD* chronic kidney disease, *SBP* systolic blood pressure, *DBP* diastolic blood pressure, *BMI* body mass index, *ESA* erythropoiesis stimulating agents, *N/A* not assessed, *NS* not significant.

**Table 3:** **Baseline laboratory values**

	Controls	CKD	CKD stage 3	CKD stages 4-5	CKD stage 5D	p value	p value
	n =559	n =2445	n =937	n =820	n =688	CKD vs controls	Between CKD groups
Creatinine (mg/dL)	0.8 ± 0.2	4.1 ± 3.3	1.6 ± 0.4	3.3 ± 1.3	8.4 ± 2.9	<0.001	<0.001
MDRD (mL/min/1.73 m^2^)	91.8 ± 16.8	N/A	43.5 ± 8.7	20.6 ± 7.5	N/A	N/A	N/A
Urea (mg/dL)	37.4 ± 9.1	101.6 ± 48.1	65.3 ± 24.3	121.9 ± 47.5	126.1 ± 42.9	<0.001	<0.001
Total cholesterol (mg/dL)	203.4 ± 35.6	178.3 ± 39.7	187.4 ± 36.5	179.1 ± 37.9	164.9 ± 42.4	<0.001	<0.001
LDL-cholesterol (mg/dL)	127.3 ± 32.8	101.8 ± 33.7	109.6 ± 31.6	101.9 ± 32.7	91.2 ± 34.8	<0.001	<0.001
HDL-cholesterol (mg/dL)	53.9 ± 15.4	49.3 ± 15.4	50.9 ± 15.4	49.2 ± 15.1	47.3 ± 15.6	<0.001	<0.001
No-HDL-cholesterol (mg/dL)	150.4 ± 36.1	129.3 ± 37.4	136.8 ± 34.5	130.6 ± 36.3	117.8 ± 39.7	<0.001	<0.001
Triglycerides (mg/dL)	115.5 ± 67.5	145.0 ± 82.3	144.7 ± 83.0	149.8 ± 85.7	139.7 ± 76.1	<0.001	NS
Glucose (mg/dL)	101.4 ± 23.6	107.6 ± 39.4	110.8 ± 37.4	108.4 ± 41.4	102.3 ± 39.2	NS	NS
Hemoglobin (g/dL)	14.5 ± 1.4	12.8 ± 1.7	13.8 ± 1.7	12.4 ± 1.4	11.9 ± 1.4	<0.001	<0.001
Transferrin (mg/dL)	283.4 ± 47.1	220.1 ± 48.8	245.9 ± 48.0	220.7 ± 42.2	193.3 ± 41.7	<0.001	<0.001
TSAT (%)	24.9 ± 12.4	27.8 ± 12.5	25.8 ± 10.5	27.3 ± 10.6	30.4 ± 15.5	0.003	<0.001
Ferritin (ng/mL)	113.8 ± 116.7	244.2 ± 308.8	170.7 ± 215.1	190.2 ± 167.7	365.5 ± 440.8	<0.001	<0.001
Uric acid (mg/dL)	5.1 ± 1.4	6.7 ± 1.6	6.8 ± 1.6	7.0 ± 1.6	6.1 ± 1.3	<0.001	<0.001
hsCRP (mg/L)	3.4 ± 7.9	4.7 ± 9.3	3.9 ± 6.1	4.8 ± 9.9	5.6 ± 11.7	<0.001	0.020
Albumin (g/dL)	4.4 ± 0.3	4.1 ± 0.4	4.2 ± 0.4	4.1 ± 0.5	3.9 ± 0.5	<0.001	<0.001
Corrected calcium (mg/dL)	9.4 ± 0.4^a^	9.2 ± 0.6	9.5 ± 0.5	9.3 ± 0.6	9.1 ± 0.7	<0.001	<0.001
Phosphorus (mg/dL)	3.5 ± 0.5^a^	4.1 ± 1.1	3.4 ± 0.6	4.1 ± 0.8	4.9 ± 1.3	<0.001	<0.001
Intact PTH (pg/mL)	N/A	181.7 ± 193.5	85.6 ± 69.0	173.5 ± 123.8	297.1 ± 273.2	N/A	<0.001
25-hydroxi-vitamin D (ng/L)	20.1 ± 7.9	16.1 ± 7.5	16.7 ± 7.8	16.2 ± 6.8	15.1 ± 7.4	<0.001	<0.001
1,25-hydroxi-vitamin D (pg/mL)	33.6 ± 13.9	16.8 ± 10.9	21.5 ± 11.2	17.4 ± 10.0	8.3 ± 5.1	<0.001	<0.001

Data expressed as mean value ± standard deviation. ^a^Data available in 36% of subjects. *Abbreviations:* *CKD* chronic kidney disease, *MDRD* glomerular filtration rate estimated with the 4-variable MDRD equation, *TSAT* transferring saturation, *PTH* parathyroid hormone, *hsCRP* high-sensitivity C reactive protein, *N/A* not assessed, *NS* not significant.

**Figure 1:**
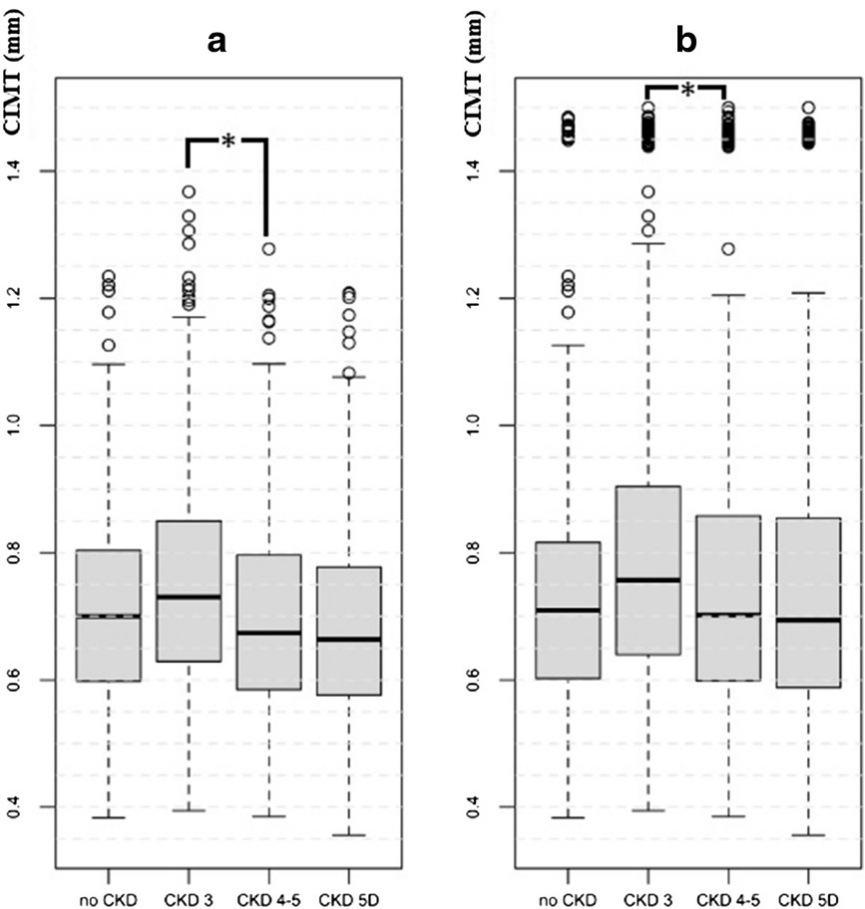
**Mean carotid intima-media thickness in controls and chronic kidney disease patients.** Distribution of carotid intima-media thickness (cIMT) in controls and in different chronic kidney disease (CKD) stages. **a)** Patients without plaques in common carotid arteries. **b)** All patients (plaques truncated at 1.5 mm). *p <0.001.

**Figure 2:**
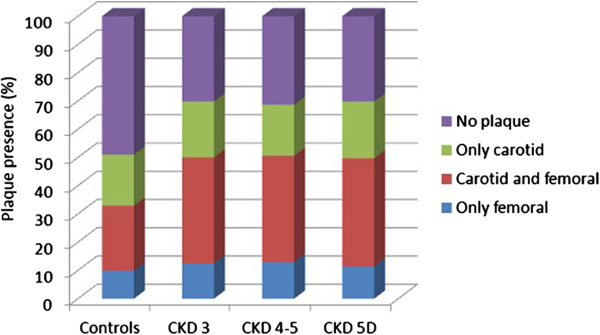
**Plaque presence in carotid and femoral ultrasound.** Prevalence and distribution of atheromatous plaques in carotid and/or femoral arteries in both controls and chronic kidney disease (CKD) patients.

**Figure 3:**
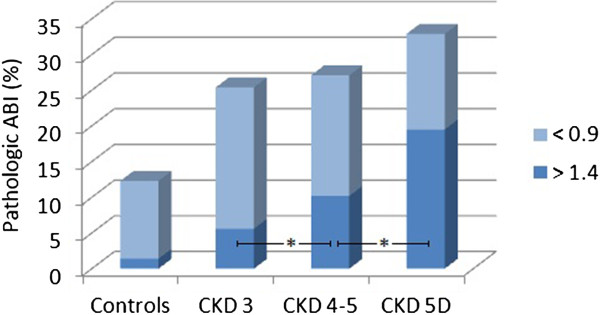
**Pathologic ankle-brachial index in controls and chronic kidney disease patients.** Prevalence of pathologic ankle-brachial index (ABI) and variation of ABI <0.9 and ABI >1.4 through chronic kidney disease (CKD) stages. *p <0.001.

Results of tests to detect subclinical atheromatosis are showed in Table 4. There was no difference between controls and CKD patients, but cIMT was lower in patients with more advanced CKD (Figure 1). Statistically significant differences were shown when a model was used in which plaques in common carotid arteries were truncated to a cIMT value of 1.5 mm.On the contrary, CKD patients presented a higher prevalence of atheromatous plaques, both globally and in carotid and femoral arteries. No differences were found between CKD stages. It is important to highlight a high rate of patients with femoral but not carotid plaques (Figure 2). Similar results were found with the measure of ABI: higher prevalence of pathologic values in CKD, with a progressive rise of prevalence in more advanced CKD. Besides, as CKD progresses, there are more patients with an ABI compatible with vascular stiffness (ABI >1.4) and lower with an ABI <0.9, compatible with distal ischemia (Figure 3).

**Table 4:** **Subclinical atheromatosis detection in CKD and non-CKD patients**

	Controls	CKD	CKD stage 3	CKD stages 4-5	CKD stage 5D	p value	p value
	n =559	n =2445	n =937	n =820	n =688	CKD vs controls	Between CKD groups
**cIMT (mm)**							
All patients^a^	0.705 (0.605-0.815)	0.725 (0.615-0.870)	0.765 (0.645-0.891)	0.705 (0.600-0.855)	0.695 (0.595-0.850)	<0.001	<0.001
Plaque-free common carotid^b^	0.710 ± 0.150	0.710 ± 0.160	0.749 ± 0.164	0.699 ± 0.157	0.689 ± 0.154	NS	<0.001
**Atheroma plaque presence (%)**							
Any territory	52.4	70.0	70.5	69.6	70.0	<0.001	NS
Only carotid artery	18.1	18.8	19.3	17.6	19.4	<0.001	NS
Only femoral artery	10.0	12.6	12.9	13.3	11.2	<0.001	NS
Carotid and femoral arteries	24.3	38.7	38.3	38.6	39.3	<0.001	NS
**ABI**							
Mean ABI	1.03 ± 0.10	1.06 ± 0.27	1.02 ± 0.19	1.05 ± 0.21	1.14 ± 0.38	NS	<0.001
Pathologic ABI (%)	12.3	28.0	25.3	27.1	32.9	<0.001	0.003
ABI >1.4 (%)	1.4	11.0	5.6	10.2	19.5	<0.001	<0.001
ABI <0.9 (%)	10.9	17.0	19.8	16.9	13.4	<0.001	0.003
**Atheromatosis score (%)**						<0.001	NS
AS 0	38.1	30.3	28.4	33.4	29.3		
AS 1	20.0	11.5	13.1	9.9	11.0		
AS 2	41.7	53.5	54.1	51.5	55.2		
AS 3	0.0	4.4	4.2	5.0	4.1		

Data expressed as percentage or mean value ± standard deviation. ^a^Values for all the patients, truncating IMT values of patients with plaques at 1.5 mm. Data expressed as median (interquartile interval). ^b^Values for patients with no plaque in any common carotid artery. *Abbreviations:* *CKD* chronic kidney disease, *cIMT* common carotid intima-media thickness, *ABI* ankle-brachial index, *AS* atheromatosis score, *NS* not significant.

Finally, AS was evaluated, with a higher rate of worse scores in CKD patients, but no significant differences between stages.

## Discussion

NEFRONA is the first large cohort study evaluating the prevalence and evolution of subclinical atheromatosis in CKD patients, aimed to define the role of non-invasive early diagnostic tests.

Cardiovascular disease is the main cause of morbidity and mortality in CKD patients, and it continues to be so despite the better knowledge on vascular disease pathogenesis and the improvement in CKD treatments [1, 21, 26]. Appropriate evaluation of cardiovascular risk is essential, and several non-invasive and easily performable methods, like vascular ultrasound, are currently under study.

In this population, we found that the CKD-group has a higher age and proportion of male subjects, and higher rates of hypertension, diabetes and dyslipidemia than the controls. However, this worse cardiovascular profile is more pronounced in earlier stages of CKD. The percentage of diabetic and vascular etiologies of CKD is proportionally lower in more advanced stages. Blood pressure values and BMI equally show this trend. These facts could be explained by an incidence-prevalence bias (or Neyman bias), since as renal dysfunction progresses, patients with worse cardiovascular health have a lower possibility of staying free of cardiovascular events, and hence, of being recruited for this study. There is probably a selection bias too, given that patients in CKD stage 5d were significantly younger, and age has been shown the most important risk factor for atheromatosis development. It is also rather remarkable the high rate of well-known cardiovascular risk factors in stage 3 CKD patients. Altogether, it seems increasingly important and evidence-based that primary prevention strategies must be started earlier in the course of the disease.

All evaluated pharmacological treatments are much more frequently prescribed in CKD patients than in controls, as it was expected. Antihypertensive, antidiabetic and hypolipidemic drugs are more common in CKD stages 4-5 than in stage 3, but in dialysis these treatments are much less frequent. This tendency is explained by the baseline differences, but also by the well-known reverse epidemiology effect in cardiovascular risk factors: dialysis patients usually have lower LDL-cholesterol levels, so they are prescribed less statins.

On the other side, treatments for control of anemia and mineral-bone disorders grow exponentially in more advanced CKD stages. Two interesting pieces of information can be highlighted: first, a very low percentage of patients treated with native vitamin D regardless of the stage, despite the usually low plasma levels and the growing evidence of the benefits of vitamin D supplementation [27–30]; second, the high rate of patients in more advanced stages of CKD receiving calcium-based phosphate binders, despite their extremely high risk of vascular calcification.

In the evaluation of laboratory information, mean values show that patients are globally well controlled, and we confirm the reverse epidemiology phenomenon in lipids (the frequently described tendency to spontaneously normalize their lipid profile in end-stage renal disease, that has been associated to more inflammation and malnutrition). It is important to highlight the low values of inflammation markers in all CKD stages. Anemia and mineral-bone disorder parameter analysis appear to show a mainly good adherence to current guidelines, at least in this subpopulation free of cardiovascular history.

cIMT is a good cardiovascular risk marker in subjects with normal renal function [31, 32]. A few studies have also proved that cIMT can predict ischemic events in CKD patients, but most of them were conducted in dialysis patients with small sample sizes [33–37]. In the NEFRONA study, we did not find any difference in cIMT between CKD patients and controls, unless plaque presence was corrected to a higher cIMT value. However, we did find that cIMT was higher in CKD stage 3, and progressively lower with more advanced stages. Again, there seems to be a survival bias involved, given the cross-sectional nature of this data, and an incidence-prevalence bias, since only patients free of prior cardiovascular events were included. It is likely that patients with more advanced CKD have a lower cIMT because those who would have had a higher cIMT probably already had a cardiovascular event. It is a well-known factor that CKD patients have a higher chance of suffering a fatal or non-fatal vascular event than eventually requiring dialysis, so those patients arriving to more advance CKD stages are usually considered “cardiovascular survivors” [21]. This fact should be proved in the prospective analysis of the NEFRONA study. More specific data about cIMT, such as different site measures or differences between left and right cIMT, is under current analysis.

Atheromatous plaque presence directly affects cardiovascular prognosis in general population [38], and it seems clearly involved in CKD morbimortality [39]. There is evidence showing the predictive effect of the presence and extent of vascular plaques on dialysis patients [40, 41]. Besides, the place where calcium is deposited is also important: it seems that intimal rather than medial calcification has a worse prognosis [42–44]. In our study, plaque prevalence is significantly higher in CKD patients than in subjects with normal renal function, but there is no difference between CKD stages. We explain this fact by the same biases as for cIMT; age seems to be the main determinant for the atheromatous load, so only younger patients with less risk factors reach stage 5 CKD free of cardiovascular events.

One interesting finding is the high rate of femoral plaques, even in patients with no carotid atheromatosis (between 10 and 12%). Some studies have started to explore the evaluation of ultrasound diagnosis of femoral plaques in general population and in other pathologies [45, 46]. In CKD, evaluation of this site has been studied by simple radiology, and it has been associated to peripheral artery disease and cardiovascular prognosis [47]. In order to perform an adequate vascular risk assessment, femoral ultrasound should complement carotid ultrasound. More extensive data on the specific characteristics of atheromatous plaques and difference between subgroups is beyond the aim of this article, and it has partially been published [48].

Peripheral artery disease, defined as a pathologic ankle-brachial index has also shown to have a predictive value over new-onset cardiovascular events in non-CKD [49, 50] and CKD subjects [51–53], but again, most data come from dialysis cohorts. In the NEFRONA study, CKD was associated with a pathologic ABI. In more advanced stages of CKD, pathologic ABI was more frequently in the higher rank (>1.4, related to severe vascular calcification) than in the ischemic range (<0.9).

Altogether, CKD patients present a higher atheromatous score than non-CKD subjects, but no difference could be found between CKD stages, probably due to the cross-sectional nature of this initial analysis and the uneven distribution of cardiovascular risk factors. Further and more complete information will be revealed when follow-up data are published.

This study has some limitations, being the first one the cross-sectional nature of these initial results. There is an intentional bias, because only patients with no history of cardiovascular events were included. This bias was necessary, but its consequences have to be considered when interpreting the results. Finally, control subjects are not perfectly matched, and also present a selection bias: they were selected from patients seeking medical attention in Primary Care centers, so they might not be a fully representative sample of the general population.

## Conclusions

NEFRONA is the first study presenting the real prevalence of subclinical atheromatosis in a large cohort of CKD patients, compared to subjects with normal renal function. As an added value, all diagnostic tests were performed by the same exploration teams, avoiding differences between observers.

The cardiovascular risk profiles and treatment policies through different CKD stages is also presented. Our results indirectly suggest that patients with more advanced renal dysfunction are those who have avoided cardiovascular events.

Subclinical atheromatosis is very prevalent in chronic kidney disease patients. Femoral atheromatous plaques are very frequent, even in patients without carotid plaques; hence, femoral ultrasound should also be performed for a correct evaluation of cardiovascular risk. Further data on the evolution and prognosis of subclinical atheromatosis will contribute to the understanding of atheromatous disease in kidney disease when the prospective analysis of the NEFRONA study is published. This initial analysis should generate new hypothesis and stimulate the study of the effect of earlier primary prevention strategies on cardiovascular morbidity in CKD patients.

## Authors’ information

Jose Manuel Valdivielso and Elvira Fernández share senior authorship.
